# Nonoperative Treatment of Finger Flexor Tenosynovitis in Sport Climbers—A Retrospective Descriptive Study Based on a Clinical 10-Year Database

**DOI:** 10.3390/biology11060815

**Published:** 2022-05-25

**Authors:** Sabrina Mohn, Jörg Spörri, Flavien Mauler, Method Kabelitz, Andreas Schweizer

**Affiliations:** 1 Division of Hand Surgery, Department of Orthopaedics, Balgrist University Hospital, University of Zurich, Forchstrasse 340, 8008 Zurich, Switzerland; kabelitz.method@gmail.com (M.K.); andreas.schweizer@balgrist.ch (A.S.); 2 University Centre for Prevention and Sports Medicine, Department of Orthopaedics, Balgrist University Hospital, University of Zurich, Forchstrasse 319, 8008 Zurich, Switzerland; joerg.spoerri@balgrist.ch; 3 Sports Medical Research Group, Department of Orthopaedics, Balgrist University Hospital, University of Zurich, Lengghalde 5, 8008 Zurich, Switzerland; 4 Department of Plastic Surgery and Hand Surgery, Kantonsspital Aarau, Tellstrasse 25, 5001 Aarau, Switzerland; flavien.mauler@gmail.com

**Keywords:** tenosynovitis, flexor tendon, finger injury, rock climbing, overuse, nonoperative

## Abstract

**Simple Summary:**

Finger flexor tenosynovitis is among the most frequent overuse injuries in sport climbers. Targeted therapy is currently based mostly on reports of the anecdotal practical experience of single centers rather than scientific investigations, as there is very little research available on this pathology. The aim of this study was to describe the nonoperative treatment outcomes of finger flexor tenosynovitis treatments in sport climbers by retrospectively asking patients about injury triggers, therapy contents and outcomes. All patients were initially treated conservatively, and only one of the patients needed further therapy in the form of a single injection with hyaluronic acid; none of them underwent further operative treatment. The average symptom duration was 30.5 weeks, and all patients were able to resume climbing, with approximately 75% of them regaining or exceeding their initial climbing level. These good to excellent outcomes and no correlation between particular therapy contents and therapy outcome suggest that finger flexor tenosynovitis in sport climbers has a favorable natural course without requiring invasive therapy. However, further cohort studies and, ultimately, randomized controlled trials are needed to conclusively confirm our promising observations from this study.

**Abstract:**

The aim of this study was to describe the nonoperative treatment outcomes of finger flexor tenosynovitis in sport climbers and to evaluate the association with baseline measures and therapy contents. Sixty-five sport climbers (49 males, mean age 34.1 years) diagnosed with tenosynovitis of the finger flexors were retrospectively asked about injury triggers, therapy contents and outcomes. Pulley thickness was measured by ultrasound. All patients were initially treated conservatively, and only one of the patients needed further therapy (single injection with hyaluronic acid); none of them underwent surgical treatment. The most frequently applied therapy was climbing-related load reduction (91%). The treatment resulted in a statistically significant reduction in pain intensity during climbing (before/after therapy ratio [Visual Analog Scale (VAS)/VAS] = 0.62, 95% CI = 0.55, 0.68). The average duration of the symptoms was 30.5 weeks (range 1–120 weeks). In a multiple linear regression analysis, initial daily life pain intensity and a climbing level higher than 7b according to the French/sport grading scale were the only predictive parameters for the relative change in pain intensity and symptom duration, respectively. All patients were able to resume climbing, with 75% regaining or even exceeding their initial climbing level. The good to excellent outcomes and no correlation between particular therapy contents and therapy outcome may suggest that finger flexor tenosynovitis in sport climbers has a favorable natural course without requiring invasive therapy. However, further cohort studies and, ultimately, randomized controlled trials are needed to conclusively confirm our promising observations.

## 1. Introduction

**Figure 1:**
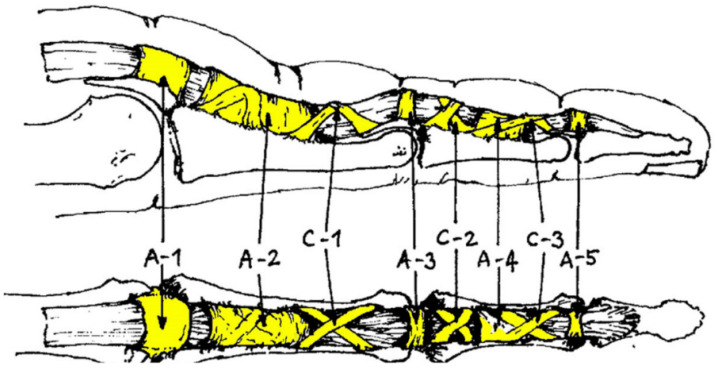
The tendon sheath is reinforced by five annular (A1–5) and three cruciform (C1–3) ligaments that hold the flexor tendon close to the bone.

Sport climbing is a sport that is attracting increasing popularity and was part of the Olympic Games for the first time in Tokyo 2021 [1,2,3]. Recent studies have shown that finger flexor tenosynovitis at the A2 (proximal phalanx) and A4 (middle phalanx) pulleys is one of the most frequent overuse injuries in rock climbers [4,5,6,7]. The pulleys arch over the tendon sheath, holding the flexor tendon close to the bone and guaranteeing its optimal function (Figure 1) [7,8]. The A2 and A4 pulleys play the most important role in the guidance of the flexor tendon [7,8].

**Figure 2:**
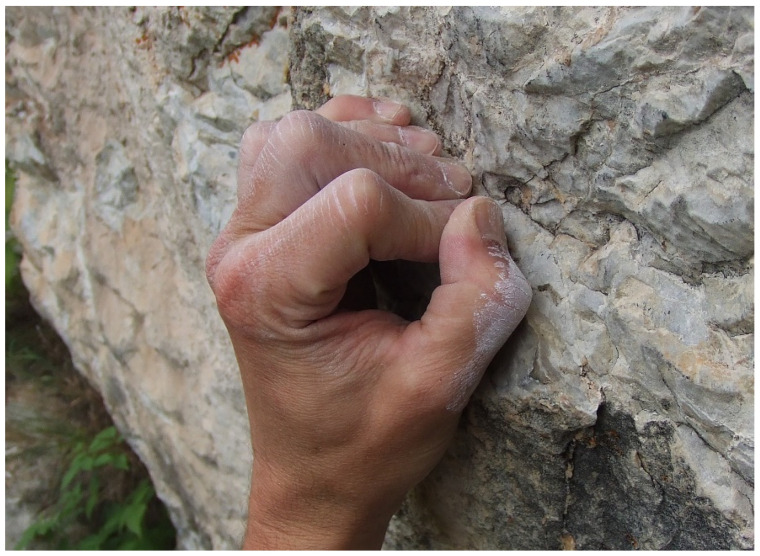
In the full crimp position, the PIP joint is fully flexed, and the DIP joint hyperextends.

The inflammatory response of the tendon sheath called tenosynovitis results from repetitive loading [4,5,7]. This occurs either acutely, after an exceptionally hard training day, or chronically after multiple training sessions [4,5,7]. Specific finger training (e.g., “campus board” training, a training tool with ladders of rungs to improve dynamic movement without using the feet), excessive dynamic movements, and constant use of the full crimp grip position (in this position, the proximal interphalangeal (PIP) joint is flexed from 90° to 100°, and the distal interphalangeal joint is hyperextended, typically used to hold small edges (Figure 2) [8]) have been associated with a higher risk of developing finger flexor tenosynovitis [9].

Affected individuals typically suffer from pain along the palmar surface of the digit [4,10] at approximately the same location as pulleys A2 (proximal phalange) and A4 (middle phalange). Accordingly, it is considered the most important differential diagnosis of a pulley injury [4]. Other differential diagnoses include PIP joint distortion with collateral ligament and palmar plate injury, complete and partial flexor digitorum superficialis (FDS) tendon avulsion or disruption or an extensor tendon lesion [2,4]. Diagnosis can be confirmed through ultrasound examination, which usually shows an accumulation of fluid around the tendon [10] as well as synovitis or scarring if the problem persists [11], both of which lead to an increased pulley thickness.

Targeted therapy is currently based mostly on reports of the anecdotal practical experience of single centers [4,7,10] rather than scientific investigations, and previous reports have focused mainly on the treatment of trigger finger tenosynovitis [12,13,14]. Accordingly, to the best of our knowledge, only one experimental study about the treatment of finger flexor tenosynovitis in sport climbers has been published to date [10]. Schöffel et al. developed a specific stage-based treatment regimen with an initial conservative approach and peritendinous corticosteroid injections for patients with persisting symptoms [10]. They reported significant pain relief and the absence of complications [10]. However, the study did not compare this approach with other conservative treatment strategies [10]. Published data also indicate that accidental intratendinous instead of peritendinous injection has been associated with complications such as tendon rupture, fat necrosis, and flare reaction [2,10].

The nonoperative clinical standard treatment of sport climber tenosynovitis usually consists of anti-inflammatory medication, external ointment application, temporary splint immobilization, brush massages or cryotherapy as well as a minimization of climbing [4,7,10]. Studies on the operative treatment of finger flexor tenosynovitis are currently lacking.

In our daily clinical work, we increasingly observed good to excellent results with the predominantly used conservative treatment approach for finger flexor tenosynovitis. Based on Schöffels study [10], however, the extent to which the pathology would heal with an exclusively noninterventive approach of treatment without any injections remains unclear. Due to the lack of scientific data on the natural course of this pathology, we decided to investigate whether conservative therapy is sufficient to support a favorable natural course or whether some cases require more invasive treatment. In the future, we should be able to inform patients with flexor tenosynovitis in more detail about the expected duration and course of symptoms as well as about their training adaptations and other therapeutic techniques to support an optimal healing process.

Consequently, the aims of this study were (1) to describe the baseline measures, injury characteristics, retrospectively perceived injury triggers, therapy contents and therapy outcomes of nonoperative finger flexor tenosynovitis treatments as part of climbing-related consultations and (2) to evaluate the association of therapy outcomes with baseline measures and therapy contents.

## 2. Materials and Methods

### 2.1. Study Design and Participants

The present study was designed as a retrospective descriptive study of patients diagnosed with tenosynovitis of the finger flexors due to sport climbing and were treated at our university hospital from 2010 to 2019. All participating patients underwent standard treatment, as further outlined below. The inclusion criteria were a consultation for finger flexor tenosynovitis in the abovementioned period of time, a minimum age of 18 years, rock-climbing anamnesis and a minimum follow-up time of 12 months after the first consultation. Patients diagnosed with pulley rupture, flexor tendon lesions, or other simultaneous finger injuries through trauma were excluded. Basic data and patient information were obtained from the local database as a retrospective chart review. All eligible individuals were contacted by a mailed questionnaire concerning the course of their injury. The sample size was determined by the inclusion of all eligible patients. This study was approved by an institutional review board and the local ethics committee (BASEC No. 2021-00274). All participants provided informed consent prior to participation.

### 2.2. Clinical and Radiographical Diagnosis

At the initial presentation, the diagnosis was performed through clinical evaluation. This consisted of painful palpation of the volar aspect of the finger, typically localized superficially to pulleys A2 and A4 of the affected digit. Furthermore, an ultrasound examination of the pulleys, tendon sheaths, and tendons was performed in a transverse plane as described previously in other articles examining these finger structures [10,15]. An increased accumulation of liquid around the tendon corresponding to the inflammatory response as well as a thickened pulley with an altered tendon structure were considered as evidence to support the diagnosis of sport climber synovitis [10,11].

### 2.3. Treatment Algorithm

**Figure 3:**
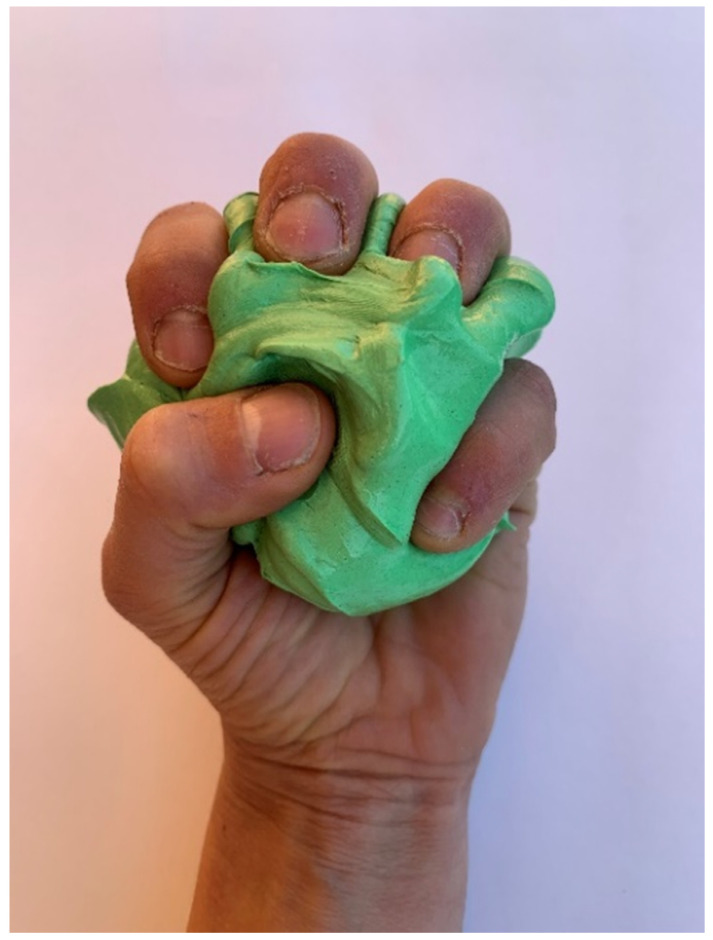
Patients received a piece of modelling clay to strengthen the flexor muscles.

**Figure 4:**
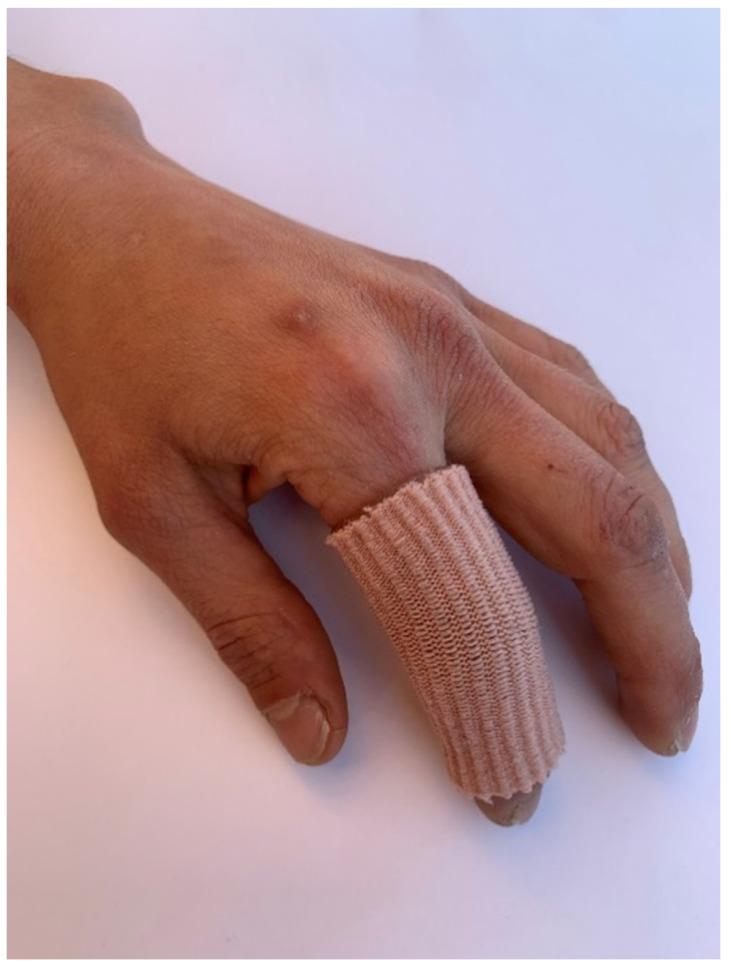
**Furthermore,** patients received a compression fingerling to decrease soft tissue swelling.

Nonoperative therapy included the use of modelling clay (Figure 3) and a silicon-made compression finger cot (Figure 4). Patients were instructed to exercise daily with the modelling clay to strengthen the flexor muscles as well as to warm up the flexor mechanism prior to rock climbing. They were advised to carry compression fingerlings during the night to decrease soft tissue swelling and to enhance lymphatic drainage. Furthermore, patients were also taught the “proper” grip technique while climbing. This included avoiding full and half crimp finger positions as much as possible and limiting climbing to the open grip positions. Thus, easy climbing with large holds on walls that do not go far beyond the vertical direction was encouraged rather than complete immobilization and rest. While finger taping was not part of the recommended therapy, many climbers independently opted for tape around the affected pulley while climbing.

No regular clinical follow-up was performed, as improvement was expected without further medical interventions. Further consultations were only carried out if symptoms remained unchanged or worsened. In rare cases where there was no long-term improvement, further therapeutic options were discussed. This comprised an injection of corticosteroids or hyaluronic acid into the tendon sheath.

### 2.4. Follow-Up Questionnaire

All patients were asked to fill out a specially designed questionnaire about the course of their sport climber’s tenosynovitis. The questionnaire contained questions about the circumstances of injury, the application of therapeutic measures and functional and sport-specific outcomes. For questions about perceived injury triggers or training attitudes, multiple answers were permitted.

To assess the pre- and posttreatment climbing level, the French grading scale was used (Table 1). As an example, a sport climbing grade of 7b represents an advanced climbing level in both men and women [16]. Approximately two to three specific training sessions per week are required to successfully climb routes at this level. The most difficult climbing level, achieved in the two years before the onset of tenosynovitis in both on-sight (free-climbing a route at first try without any prior information) and redpoint styles (free-climbing a route after practicing it at least once before), was considered the patient’s initial climbing level. This was then compared to the highest level of climbing route completed within one year of completing therapy.

Pain during or after climbing or during daily life was evaluated before and after treatment using the VAS with scores from 1–10. With respect to range of motion (ROM), participants were asked to roughly compare the full flexion and extension of the affected finger(s) with the corresponding nonaffected finger(s) of the contralateral side. A similar comparison was made by asking about any remaining subjective differences in finger strength. In addition, participants were asked to rate the overall functional outcome function using the Single Assessment Numeric Evaluation (SANE), which has been previously validated as a reasonable measure for patients undergoing common hand procedures [17].

Some questionnaires were returned with missing answers, which is why the total number of cases in the results section varies for some of the parameters.

### 2.5. Radiographic Analysis

**Figure 5:**
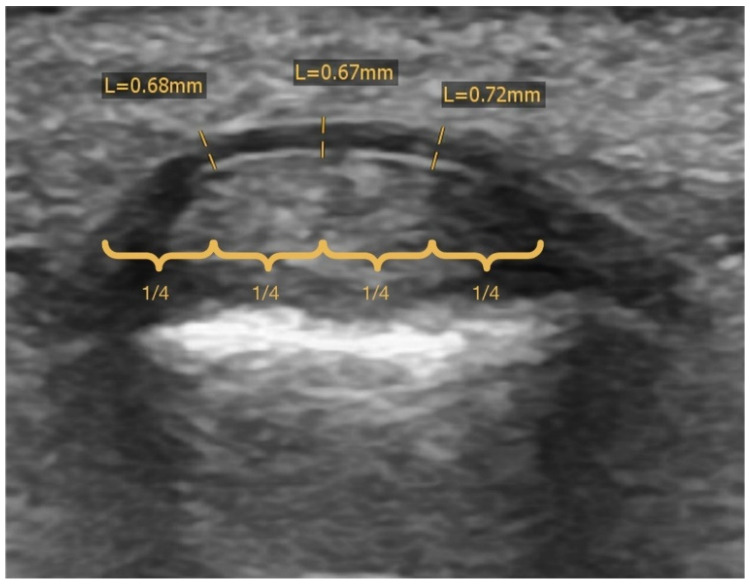
Pulley width was measured in a transverse plane (mm) at approximately 25%, 50% and 75% of the transverse pulley diameter to determine the average value.

The ultrasound evaluation performed during the initial consultation was analyzed. The width of the pulley was measured as rectangular to its surface at approximately 25%, 50% and 75% of its transverse diameter (Figure 5) to determine the average value.

### 2.6. Statistical Analysis

The statistical analysis was performed as follows: (1) all baseline measures, injury characteristics, retrospectively perceived injury triggers, therapy contents and therapy outcomes were reported as the mean with 95% confidence intervals (CI) or percentage proportion, respectively; (2) all interval scaled data were assessed for normal distribution using graphical techniques (i.e., histograms and quantile-quantile plots) and shape parameters (i.e., skewness and kurtosis coefficients) [18]. Skewness values of <0.4 and kurtosis values of <0.8 were markedly below the common reference boundaries for substantial departure from normality (skewness > 2 and kurtosis > 7 according to West et al. [19]), which is why a normal distribution was assumed. (3) To evaluate the association of the therapy outcomes “relative change in perceived pain intensity” (i.e., the *before/after therapy ratio in pain intensity during climbing*) and *symptom duration* with the baseline measures *climbing years before therapy*, *average pulley thickness*, *climbing level higher than 7b*, and perceived *pain intensity in daily life and during climbing before therapy*, as well as the therapy contents *modelling clay*, *compression fingerling*, *finger stretching*, *ergotherapy*, *medication*, *taping*, and *climbing-related load reduction*, multiple regression models (stepwise method) were calculated (*p* < 0.05). For the statistical analysis, SPSS Statistics software (Version 26, IBM, New York, NY, USA) was used.

## 3. Results

### 3.1. Baseline Characteristics

Out of the 72 patients who fulfilled the predefined inclusion criteria, three patients could not be contacted, and four patients refused to complete the follow-up questionnaire. Accordingly, 65 patients (49 males and 16 females) with a mean age of 34.1 years at the time of injury served as the basis for data analysis in the present study (unless otherwise indicated in Table 2, Table 3, Table 4 and Table 5). Further baseline characteristics are summarized in Table 1. Most importantly, before treatment, the vast majority (95.4%) experienced pain during or after climbing, with a mean VAS score of 5.9. Pain during daily life was reported in 92.3% of the patients with lower intensities (mean VAS of 3.1).

### 3.2. Injury Characteristics, Perceived Injury Triggers and Training Attitudes

Detailed information about the injury characteristics, retrospectively perceived injury triggers and training attitudes are presented in Table 3. Eighteen patients (27.7%) had more than one affected pulley at the initial consultation time when diagnosis was made, with a total of 93 affected pulleys. One patient who presented with two affected pulleys at different times was considered as representing two cases. The injury exclusively occurred in the middle or ring finger (58.5% and 55.4%, respectively), except for in one case where the fifth digit was affected. Most cases were reported in the A2 pulley (84.6%), followed by the A4 pulley (23.1%). Concerning the perceived causes of pulley inflammation, hard and intense training in general was the most frequently mentioned trigger (63.1%). Further perceived triggers were repetitive attempts to perform a single hard move (41.5%) and overuse of the full crimp grip position (30.8%). When asked about their training habits, 43.1% of patients reported incorporating hang board exercises (wooden board with a variety of holds to train finger strength in different grip positions) into their training. Additional common habits included campus board exercises (32.3%), specialized training on small holds (33.8%), and more than three training and/or climbing sessions per week (30.8%).

### 3.3. Content and Outcome of Therapy

A description of the therapy content outcome is provided in Table 4. The most frequently applied therapy was climbing-related load reduction (90.8%), as 61.5% stopped climbing completely for a mean time of 4.4 weeks. Further frequent therapy modes were exercises with the modelling clay (75.4%), followed by taping (64.6%), even though this had not been recommended during the consultation, compression fingerling (35.4%), finger stretching (32.3%), oral medication (13.8%) and ergotherapy (12.3%). Only one of the patients needed further therapy in the form of a single injection with hyaluronic acid; none of them underwent further operative treatment.

The average duration of symptoms was 30.5 weeks (range 1–120 weeks). Half of the patients (50.8%) described an undulating, slow improvement, and 38.5% reported a consistent amelioration. The relative reduction in climbing-associated pain intensity after therapy was 38% on average. Nine patients (13.8%) were completely pain free, 51 patients (78.5%) felt occasional or rare pain, and the remaining five patients (7.7%) still experienced frequent pain.

All patients were able to resume climbing, with approximately three-quarters regaining or even exceeding their initial climbing level (both redpoint and on-sight style) within one year following the end of therapy (76.9% and 78.5%). None of the patients reported any remaining severe limitation of strength or ROM compared to the same finger on the contralateral side. Overall, a mean SANE value of 93% was found, implying a very good global outcome. Accordingly, more than nine out of ten patients rated the therapy procedure as very good or good. When asked about long-term changes in behavior beyond therapy, the most frequently given answers were paying more attention to the warm-up (41.5%) and using the full crimp grip position less frequently (26.2%).

A multiple linear regression analysis evaluating the association of the therapy outcomes “relative change in perceived pain intensity during climbing” and symptom duration with baseline characteristics and therapy components is shown in Table 5. The initial pain intensity in daily life was found to be a significant predictor of the relative reduction in pain during climbing through nonoperative treatment and accounted for 12% of its variance. Regarding symptom duration, an initial climbing level of higher than 7b was a significant determinant, explaining 9.2% of the variance. Neither one of the individual therapy contents nor the sonographically assessed average pulley thickness or the reduction in the climbing-related load was revealed to have an influence on the therapy outcome.

## 4. Discussion

The main findings of the study were as follows: (1) all patients were treated conservatively without surgery, and only one patient required further invasive therapy; (2) the applied treatment resulted in a statistically significant reduction in pain intensity during climbing (before/after therapy ratio [VAS/VAS] = 0.62, 95% CI = 0.55, 0.68); (3) the mean symptom duration was 30.5 weeks, with a tendency toward an undulating, slow improvement (50.8%); (4) although most patients experienced remaining rare or occasional low-level pain, they were able to resume full load climbing, and 75% regained their initial climbing grades or higher after treatment; and (5) in a multiple linear regression analysis, initial daily life pain intensity and a climbing level of higher than 7b according to the French/sport grading scale were the only predictive parameters for the relative change in pain intensity and symptom duration, respectively.

### 4.1. Injury Characteristics and Etiology

The most typical injuries observed were at the A2 and A4 pulleys of the index and middle fingers. These findings are supported by other pulley injuries, such as ruptures predominantly affecting the same locations [4,5,7]. In the same context, z biomechanical analysis during climbing showed that the highest forces are applied to these structures, especially when using the full crimp grip position [2,5,7,20]. Hard and intense training, the extensive use of the full crimp grip position and repetitive attempts of a single hard move were perceived as the main injury triggers in our study. This suggests that tenosynovitis is a consequence of chronic stress rather than acute trauma, as proposed earlier [2,4,7].

### 4.2. Treatment and Outcome

As previous articles mainly focused on pulley ruptures, there are very limited data on the treatment and outcome of finger flexor tenosynovitis in rock climbers. Studies on the operative treatment of finger flexor tenosynovitis are currently lacking. However, the literature on the treatment of pulley ruptures indicates that surgical pulley reconstruction is reserved for Grade IV injuries with multiple ruptures because it is associated with prolonged sports rest, the risk of stiffness and poor functional outcomes if the gliding of the tendon is impaired [21,22,23,24]. Furthermore, encircling reconstruction techniques bear the risk of cortex atrophy of the proximal phalanx [21,23]. General perioperative complications include protracted wound healing, postoperative infections, the risk of scar tissue formation or intraoperative neurovascular lesions [25]. Additionally, the postoperative necessity of monthly ergotherapeutic treatment should not be neglected [25].

Regarding finger taping, which is a widely used approach among sport climbers to treat finger injuries in practice, previous studies demonstrated that this specific intervention has a limited effect when the pulley is still intact [9,26,27]. Thus, while taping is recommended after a pulley rupture [27,28,29], climbers who use this method purely as a preventive method were found to be even more susceptible to finger injury [30,31]. However, studies on using taping as a conservative therapy approach in the treatment of finger flexor tenosynovitis in sport climbers are lacking to date.

In their stage-related treatment regimen, Schöffel et al. reported a relative pain reduction of 84.2% after the second injection, with 57.5% of all patients being completely pain free while climbing [10]. All patients were able to resume climbing with full load after a mean time of 32.9 days after the first injection, and only one patient did not regain their initial climbing grade [10]. No local complications due to corticosteroid injections occurred [10]. Thus, Schöffel et al. argue that injection therapy is justified in cases where the initial conservative management fails after four weeks or patients initially present with symptoms persisting longer than six weeks [10]. However, the general risks of injections, such as tendon rupture, fat atrophy and local infection, must be considered, although limited data exist on these complications [32]. In particular, perioperative corticosteroid injections followed by trigger finger release surgery significantly increased the rate of deep infections [32].

Comparing our findings to the results of Schöffel et al., first, the different treatment approaches must be noted. Whereas all but one of our patients were treated exclusively conservatively, all of Schöffel’s patients received a corticosteroid injection. Bearing this difference in mind, a slightly lower relative pain reduction, a higher proportion of residual low-level pain and a longer symptom duration were observed. However, a direct comparison with the latter is difficult, because they only described symptom duration in terms of 73.8% of all climbers being completely pain free after the second injection at a mean of 20.9 ± 23.1 days without providing a mean symptom duration of all patients [10]. Nevertheless, similar functional and sport-specific outcomes and a lower risk of complications than after injection therapy suggest that a longer conservative attempt is justified when treating finger flexor tenosynovitis in climbers.

The average outcome of nonoperative finger flexor tenosynovitis treatment seems to be largely dependent on the patients’ initial pain intensity before the therapy as well as their climbing level. In contrast, in the average case, the exact content of the therapy and climbing-related load reduction in general do not seem to play a decisive role. In this context, initial daily life pain intensity and a climbing level of higher than 7b were found to be the only predictive parameters for the relative change in perceived pain intensity during climbing and symptom duration, respectively.

A higher level of difficulty may imply a higher average load during climbing, which in turn could mean a higher susceptibility to continued inflammation in the stressed structures and thus, a longer symptom duration. For the relative reduction in climbing-associated pain, the initial pain intensity in daily life, but not during climbing, was the only significant determinant. This could be because pain in daily life is probably more sensitive to a more severe manifestation of tenosynovitis, which is associated with a higher initial VAS score and therefore may result in greater relative pain reduction. Although none of the individual therapy components significantly predicted either symptom duration or a reduction in climbing-associated pain, the latter was still found to be significantly increased after therapy. These findings suggest that the individually chosen therapy was successful but that no general recommendations on therapeutic interventions can be derived. In particular, a complete break from the sport is not advisable. Even a climbing-related load reduction, which is often recommended, seems to play only a minor role in therapy. Consequently, it can be assumed that finger flexor tenosynovitis in sport climbers generally shows an undulating course over several months, which is largely independent of a particular therapy mode.

### 4.3. Limitations

Several potential shortcomings of the study should be noted when interpreting the study findings. First, the sample size of this study may be deemed limited. The sample size of this study was determined by including all eligible patients who were treated at our clinic during the past 10 years, met the inclusion criteria and were willing to participate in the study. Because our clinic is one of the leading national orthopedic institutions and is well recognized in the climbing community, the resulting 10-year database of 65 climbers with flexor tendonitis can nevertheless be considered as a highly representative sample. Second, the underlying explorative questionnaire was designed specifically for the purpose of this descriptive study and has not yet been validated. Therefore, the preliminary results of the current study must be interpreted with caution. Third, the questionnaire was completed retrospectively after a considerable amount of time ranging from one to eleven years. Therefore, the answers may be affected by a certain amount of recall bias. Furthermore, since some questionnaires were returned with missing answers, there is the risk of nonresponse bias. Fourth, the ultrasound evaluation has a risk for measurement bias since there are no reported data available on the interrater reliability of the examination technique used. Fifth, the outcome assessment did not include any exact measurements of strength and ROM or a radiographical follow-up examination to evaluate a potentially reduced pulley width. Sixth, although conservative treatment was recommended in the present study, it was subsequently neither enforced nor controlled. Therefore, despite retrospectively reported adherence to a particular treatment instruction, it is possible that some quantitative and qualitative deviations from the instructed method may have occurred, as is typical for interventions in real clinical settings. Seventh, as a retrospective descriptive study, there was no comparison to a control group who received further invasive treatment, such as injection therapy or surgical treatment, by default. Although a control group would certainly have increased the quality of evidence of the study, the observation of a positive outcome in all cases (as underlying this study) may well be considered of high clinical relevance. All participating patients were able to climb again without any operative treatment, and only one of them underwent further invasive treatment in the form of an injection of hyaluronic acid. Moreover, as outlined in the introduction, there are very few scientific studies on the subject, and patient education and treatment in everyday clinical practice are largely guided by clinical experience rather than scientific evidence. This makes the evaluation of long-term clinical databases, as in the present investigation, despite its nature as a retrospective descriptive study, all the more relevant to clinical practice. Nevertheless, in view of the aforementioned major limitations of this preliminary study, further validation studies, prospective cohort studies and randomized controlled trials comparing the natural history of the condition with different available treatment strategies are needed. Only then can our promising observations from this study be considered conclusive, and the best approach to therapy can be derived.

## 5. Conclusions

The good to excellent outcomes in all our patients and no correlation between particular therapy contents and symptom duration or reduction in pain intensity suggest that finger flexor tenosynovitis in sport climbers has a favorable natural course without the requirement of invasive therapy. In the future, patients with this frequently occurring overuse injury can be informed in more detail of the following: they can expect an average symptom duration of approximately seven months (30.5 weeks) as well as an undulating course of symptoms. Moreover, they have a very good prognosis, which is largely independent of the nonoperative therapy described before. In particular, a complete break from the sport showed no benefit, so they could be reassured to continue climbing if desired.

## Figures and Tables

**Table 1:** The French/sport grading scale compared to other popular grading scales in climbing (in accordance with Draper et al., 2015 [16]).

Climbing Group		IRCRA	YDS	French/Sport	UIAA
Lower Grade (Level 1) Male and Female		1	5.1	1	I
		2	5.2	2	II
		3	5.3	2+	III/III+
		4	5.4	3-	III+/IV
		5	5.5	3	IV/IV+
		6	5.6	3+	IV/V-
		7	5.7	4	V-/V
		8	5.8	4+	V+
		9	5.9	5	VI-
Intermediate (Level 2) Male	Intermediate (Level 2) Female	10	5.10a	5+	VI
		11	5.10b	6a	VI+
		12	5.10c	6a+	VII-
		13	5.10d	6b	VII-/VII
		14	5.11a	6b+	VII/VII+
	**Advanced (Level 3)** **Female**	15	5.11b	6c	VII+/VIII-
		16	5.11c	6c+	VIII-
		17	5.11d	7a	VIII
**Advanced (Level 3) Male**		18	5.12a	7a+	VIII/VIII+
		**19**	**5.12b**	**7b**	**VIII+/IX-**
		20	5.12c	7b+	IX-/IX
	Elite (Level 4) Female	21	5.12d	7c	IX
		22	5.13a	7c+	IX+
		23	5.13b	8a	IX+/X-
Elite (Level 4) Male		24	5.13c	8a+	X-/X
		25	5.13d	8b	X/X+
		26	5.14a	8b+	X+
	Higher Elite (Level 5) Female	27	5.14b	8c	XI-
**Higher** Elite (Level 5) Male		28	5.14c	8c+	XI-/XI
		29	5.14d	9a	XI/XI+
		30	5.15a	9a+	XI+/XII-
		31	5.15b	9b	XII-
		32	5.15c	9b+	XII

IRCRA: International Rock-Climbing Research Association. YDS: Yosemite Decimal System. UIAA: Union Internationale des Associations d’Alpinisme. Bold is necessary to highlight the example of a 7b Climber as it is mentioned in the text.

**Table 2:** Baseline characteristics.

	Overall (*N* = 65)	Female (*N* = 16)	Male (*N* = 49)
age [year]	34.1 (32.0, 36.2)	33.3 (28.9, 37.7)	34.4 (32.0, 36.8)
climbing years before therapy [year]	11.1 (8.9, 13.2) ^c^	7.7 (4.4, 11.0) ^b^	12.1 (9.5, 14.7) ^b^
average pulley thickness [cm]	1.06 (0.95, 1.17) ^h^	0.97 (0.70, 1.25) ^d^	1.08 (0.92, 1.25) ^g^
climbing level higher than 7b (redpoint) [%]	63.1 ^a^	56.3 ^a^	65.3 ^a^
pain intensity during climbing before therapy [VAS]	5.9 (5.4, 6.4) ^f^	6.1 (4.9, 7.4) ^b^	5.8 (5.1, 6.5) ^e^
pain intensity in daily life before therapy [VAS]	3.1 (2.7, 3.5) ^f^	3.9 (2.7, 5.1) ^b^	2.8 (2.3, 3.3) ^e^

All data are expressed as the mean with 95% confidence intervals (CI) in brackets, unless otherwise indicated. ^a^ data reported as percentage proportion; ^b^ unknown in 1 subject (6.25% of all females, 2.04% of all males); ^c^ unknown in 2 subjects (3.07% of all patients); ^d^ unknown in 3 subjects (4.62% of all patients, 18.75% of all females); ^e^ unknown in 4 subjects (6.15% of all patients, 8.16% of all males); ^f^ unknown in 5 subjects (7.69% of all patients); ^g^ unknown in 6 subjects (12.24% of all males); ^h^ unknown in 9 subjects (13.85% of all patients).

**Table 3:** Finger flexor tenosynovitis: injury characteristics, retrospectively perceived injury triggers and training attitudes.

Hands	
dominant hand affected [%]	50.8
nondominant hand affected [%]	60.0
Digit	
digit III affected [%]	58.5
digit IV affected [%]	55.4
digit V affected [%]	1.5
Pulley	
A2 pulley affected [%]	84.6
A4 pulley affected [%]	23.1
multiple pulleys affected [%]	27.7
Perceived Injury Triggers	
hard and intensive training [%]	63.1
extensive crimping [%]	30.8
extensive use of pockets [%]	6.2
foot slipped off [%]	13.8
repeated attempts of a hard and/or dynamic single pull [%]	41.5
other [%]	16.9
Training Attitudes	
regular warm-up [%]	41.5
training on campus board [%]	32.3
training on hang board [%]	43.1
specific training on small holds [%]	33.8
more than 3 training and/or climbing sessions per week [%]	30.8
dynamo training [%]	20.0

All data are expressed as percentage proportions unless otherwise indicated.

**Table 4:** Therapy content and therapy outcome.

Therapy Content	
modelling clay [%]	75.4
compression fingerling [%]	35.4
finger stretching [%]	32.3
ergotherapy [%]	12.3
medication [%]	13.8
taping [%]	64.6
climbing-related load reduction [%]	90.8
Therapy Outcome	
symptom duration [weeks]	30.5 (24.3, 36.8) ^a,b^
before/after therapy ratio in pain intensity during climbing [VAS/VAS]	0.62 (0.55, 0.68) ^a,c^

All data are expressed as percentage proportions, unless otherwise indicated. ^a^ Data reported as the mean with 95% confidence intervals (CI) in brackets; ^b^ unknown in 5 subjects (7.69% of all patients); ^c^ unknown in 5 subjects (7.69% of all patients). VAS: visual analog scale.

**Table 5:** Multiple regression analyses evaluating the association of the therapy outcomes “relative change in perceived pain intensity” (i.e., the before/after therapy ratio during climbing) and symptom duration with the baseline measures climbing years before therapy, average pulley thickness, climbing level higher than 7b, and perceived pain intensity in daily life and during climbing before therapy, as well as the therapy including modelling clay, compression fingerling, finger stretching, ergotherapy, medication, taping, load reduction climbing and load reduction at work.

Model Parameter	Dependent Variable	Predictor	*B*	*SE_B_*	*β Weight*	*p Value ^a^*
***Model 1 ^b^*** *F = 6.613, p = 0.014, Adjusted R^2^ = 0.120, Cohen f^2^ = 0.140* *n = 42*	before/after therapy ratio in pain intensity during climbing [VAS/VAS]	pain intensity in daily life before therapy [VAS]	0.060	0.023	0.377	0.014 *
		* **excluded variables by stepwise method** *				
		*climbing years before therapy [year]*	-	-	-	0.769
		*average pulley thickness [cm]*	-	-	-	0.296
		*climbing level higher than 7b (redpoint) [0;1]*	-	-	-	0.247
		*pain intensity during climbing before therapy [VAS]*	-	-	-	0.057
		*therapy content: modelling clay [0;1]*	-	-	-	0.315
		*therapy content: compression fingerling [0;1]*	-	-	-	0.702
		*therapy content: finger stretching [0;1]*	-	-	-	0.781
		*therapy content: ergotherapy [0;1]*	-	-	-	0.822
		*therapy content: medication [0;1]*	-	-	-	0.987
		*therapy content: taping [0;1]*	-	-	-	0.286
		*therapy content: climbing-related load reduction [0;1]*	-	-	-	0.846
***Model 2 ^b^*** *F = 4.642, p = 0.037, Adjusted R^2^ = 0.082, Cohen f^2^ = 0.089* *n = 42*	symptom duration [weeks]	climbing level higher than 7b (redpoint) [0;1]	−15.821	7.343	−0.332	0.037 *
		* **excluded variables by stepwise method** *				
		*climbing years before therapy [year]*	-	-	-	0.906
		*average pulley thickness [cm]*	-	-	-	0.447
		*pain intensity during climbing before therapy [VAS]*	-	-	-	0.206
		*pain intensity in daily life before therapy [VAS]*	-	-	-	0.282
		*therapy content: modelling clay [0;1]*	-	-	-	0.144
		*therapy content: compression fingerling [0;1]*	-	-	-	0.244
		*therapy content: finger stretching [0;1]*	-	-	-	0.580
		*therapy content: ergotherapy [0;1]*	-	-	-	0.168
		*therapy content: medication [0;1]*	-	-	-	0.904
		*therapy content: taping [0;1]*	-	-	-	0.176
		*therapy content: climbing-related load reduction [0;1]*	-	-	-	0.301

^a^ Level of significance: * *p* < 0.05. ^b^ Listwise deletion of missing values. VAS: visual analog scale.

## Data Availability

The data presented in this study are available on request from the corresponding author. The data are not publicly available due to privacy reasons.
